# The heterocyclic compound Tempol inhibits the growth of cancer cells by interfering with glutamine metabolism

**DOI:** 10.1038/s41419-020-2499-8

**Published:** 2020-05-04

**Authors:** Shuangyan Ye, Pengfei Xu, Mengqiu Huang, Xi Chen, Sisi Zeng, Qianli Wang, Jianping Chen, Keyi Li, Wenwen Gao, Ruiyuan Liu, Jingxian Liu, Yihao Shao, Hui Zhang, Yang Xu, Qianbing Zhang, Zhuo Zhong, Zibo Wei, Jiale Wang, Bingtao Hao, Wenhua Huang, Qiuzhen Liu

**Affiliations:** 1 Cancer Research Institute, Guangdong Provincial Key Laboratory of Cancer Immunotherapy, Guangzhou Key Laboratory of Tumor Immunology Research, School of Basic Medical Sciences, Southern Medical University, Guangzhou, China; 2 School of Biomedical Engineering, Southern Medical University, Guangzhou, China; 3 The First Affiliated Hospital of Jinan University, Guangzhou, China; 4 Zhongshan School of Medicine, Sun Yat-Sen University, Guangzhou, China; 5 Guangzhou Hospital of integrated Traditional and West Medicine, Guangzhou, China; 6 Center for medical transformation, Shunde Hospital, Southern Medical University, Foshan, China; 7 National Key Discipline of Human Anatomy, School of Basic Medical Sciences, Southern Medical University, Guangzhou, China; 8 Department of Human Anatomy, School of Basic Medical Sciences, Guangdong Medical University, Guangzhou, China

**Keywords:** Cancer metabolism, Metabolomics

## Abstract

Tempol (4-hydroxy-2,2,6,6-Tetramethylpiperidine-1-oxyl, TPL), a nitroxide compound, inhibits proliferation and increases the vulnerability of cancer cells to apoptosis induced by cytotoxic agents. However, the molecular mechanism of TPL inhibiting cancer cell proliferation has not been fully understood. In this study, we evaluated the metabolic effect of TPL on cancer cells and explored its cancer therapeutic potential. Extracellular flow assays showed that TPL inhibited cellular basal and maximal oxygen consumption rates of mitochondrial. ^13^C metabolic flux analysis showed that TPL treatment had minimal effect on glycolysis. However, we found that TPL inhibits glutamine metabolism by interfering with the oxidative tricarboxylic acid cycle (TCA) process and reductive glutamine process. We found that the inhibitory effect of TPL on metabolism occurs mainly on the step from citrate to α-ketoglutarate or vice versa. We also found that activity of isocitrate dehydrogenase IDH1 and IDH2, the key enzymes in TCA, were inhibited by TPL treatment. In xenograft mouse model, TPL treatment reduced tumor growth by inhibiting cellular proliferation of xenograft tumors. Thus, we provided a mechanism of TPL inhibiting cancer cell proliferation by interfering with glutamine utilization that is important for survival and proliferation of cancer cells. The study may help the development of a therapeutic strategy of TPL combined with other anticancer medicines.

## Introduction

Tempol (4-hydroxy-2,2,6,6-Tetramethylpiperidine-1-oxyl, TPL), a stable nitroxide radical used as a contrast agent in magnetic resonance imaging^1^, has been reported to induce cellular apoptosis in many types of cancer cells^2–4^. The toxicity effect of TPL presents a concentration-dependent manner and is preferential to target cancer cells^5^. TPL induces the expression of the cycling-dependent kinase inhibitor p21waf1/cip1, and activates caspase-3 and Bax/Bcl-2 pathway^2,6^. And the cytotoxicity of TPL also depends on the induction of cellular oxidative stress^7,8^. It is reported that high concentration or prolonged TPL treatment increases cellular ROS level, while low concentration of TPL reduces ROS (ref. ^9^), which is related to the fact that TPL can switch between oxidation and reduction states, hydroxylamine and oxoammonium cation^10^. The dosage of TPL used in vivo usually mimics the MnSOD activity and plays an anti-oxidative role^11,12^. Some studies showed that TPL functions as an antioxidant and improves the performance in animal models of many diseases, including hypertension^13^, diabetes^14^, diabetic kidney disease^15^, neurodegenerative^16,17^, and ischemia–reperfusion injury^18^. Therefore, the concentration of TPL in vivo is usually too low to induce oxidative stress. So whether the in vivo TPL treatment inhibits tumor growth is still a question.

A common feature of cancer cell metabolism is the ability to use intermediates of glycolysis/tricarboxylic acid (TCA) cycle for biosynthesis and NADPH production, which are essential for building new biomass^19,20^. It’s known that cancer cells have a high-rate glucose uptake and metabolism to meet cell growth and viability. It has been reported that antioxidant, vitamin C, can disturb glucose metabolism in primary rat adipocytes^21^. But whether the antitumor effect of TPL is associated with the regulation of glucose metabolism is yet to be determined.

Aside from glucose, recent studies showed that amino acids, especially glutamine, are also involved in the biosynthesis in cancer cells^22,23^. Many types of cancer cells display elevated glutamine flux, and even some cancer cell lines exhibit glutamine addiction^24,25^. Glutamine metabolism occurs mainly in mitochondrial. Studies suggest that TPL is accumulated in mitochondrial and interacts with transition metal ions in catalytic centers of enzyme complexes associated with the respiratory chain complexes (complexes I, II, and IV), which impairs OXPHOS and reduces intracellular glutathione pool, and then induces oxidative stress and cellular apoptosis^7,26–28^. But whether TPL is involved in glutamine metabolism remains unclear.

Here, we characterized the antiproliferative effects of TPL with in vitro and in vivo experiments, and elucidated the metabolic mechanisms involved in glucose and glutamine metabolism alteration in TPL-treated ovarian cancer cells by ^13^C metabolic flux analysis. We found that TPL treatment interfered with the glutamine utilization in cancer cells. It was described below.

## Materials and methods

### Chemicals and reagents

TPL was purchased from Adamas Reagent Co., Ltd. (Shanghai, Chian). 3-(4,5-Dimethylthiazol-2-yl)-2,5-diphenyltetrazoliumbromide (MTT) and dimethyl sulfoxide (DMSO) were purchased from Sigma (St. Louis, MO, USA). Other reagents were obtained from the following companies: methanol (Kermel, Tianjin, China), chloroform, hexane, and pyridine (Aladdin, Shanghai, China), [^13^C-U_6_] glucose, [^13^C-U_5_] glutamine (Cambridge, Boston, MA, USA), 2% methoxylamine hydrochloride, N-tert-butyldimethylsily-N-methyltrifluoroacetamide (MTBSTFA), tert-butyldimethylchlorosilane (tBDMS; Sigma, St. Louis, MO, USA), glucose-free DMEM medium, and glutamine-free RPMI-1640 medium (Gibco, Waltham, MA, USA). Glucose, oligomycin, 2-DG, carbonyl cyanide 4-(trifluoromethoxy)phenylhydrazone (FCCP), and rotenone-antimycin A (Agilent Technologies, Santa Clara, CA, USA). L-glutamine and pyruvate (Thermo Fisher Scientific, Waltham, MA, USA).

### Cell culture

The human ovarian cancer line SKOV3 were obtained from ATCC. SKOV3 was maintained in RPMI-1640 medium with 10% FBS and 1% antibiotics mixture. The cells were maintained under standard culture conditions at 37 °C with a 5% CO_2_ atmosphere in a humidified incubator unless otherwise mentioned. And cells were continuously cultured in without pen/strep exposure.

### Cytotoxicity assays

MTT assays were performed to determine the viability of cell lines. Cells were seeded onto 96-well plates, allowed to attach, and grow for 12–24 h, and subsequently exposed to TPL in different concentrations or not. After 48 h, 100 μl of MTT (0.5 mg/ml in medium) was added to cells for 3 h at 37 °C. Moved MTT and slightly rinsed twice with PBS. The formazan crystals then were solubilized in 100 μl of DMSO. The samples were shaken for 15 min at 37 °C and the absorbance was read at 490 nm using Bio-Tek multi-mode reader.

### ROS level analysis

To detect the ROS level in culture cell, 35 × 10^4^ cells/well were seeded onto six-well plates, and subsequently exposed to TPL in different concentrations for 24 h after incubating at 37 °C for 12 h. Collected and washed cells twice with normal temperature PBS for ROS determination. The determination of ROS was detected using ROS Assay Kit according to the manufacturer’s instructions (Beyotime, Shanghai, China). Briefly, 1 × 10^7^ cells/ml were suspended on serum-free medium containing 10 μm/ml DCFA-DA and incubated at 37 °C in the dark for 20 min. Unincorporated DCFA-DA was moved by washing the cells with phenol red free medium. Then cells were resuspended with PBS, and immediately subjected to flow cytometric analysis. The experiments were performed at least three times.

### NAD^+^ and NADH measurement

To assess the NAD^+^ and NADH, 35 × 10^4^ cells/well were seeded onto six-well plates. The amount of NAD^+^ and NADH was measured using NAD^+^/NADH Assay Kit (Beyotime, Shanghai, China), according to the manufacturer’s protocol. Before detecting NADH, samples were heated in water bath to 60 °C for 30 min to decompose NAD^+^. Then the decomposed sample was reacted and measured at 450 nm using Bio-Tek multi-mode reader. The content of NADH in the sample can be calculated from standard curve.

### Glucose uptake analysis

To assess the glucose uptake, 6 × 10^4^ cells/well were seeded onto 12-well plates. After cells attached on the wells, exchanged the medium into medium containing 0.8 mM TPL or not to culture for 24 h. The measurement of glucose was detected using Glucose Colorimetric Assay Kit II according to the manufacturer’s instructions (BioVision, SanFrancisco, CA, USA). Briefly, 2 μl of cell culture supernatant was added to 48 μl of glucose assay buffer in 96-well plate, and mixed well. Added 50 μl of reaction mix, containing 46 μl glucose assay buffer, 2 μl glucose enzyme mix, and 2 μl glucose substrate mix, to each well containing the test samples. Incubated the reaction for 30 min at room temperature (RT) that protected from light. Measured absorbance at 450 nm in a microplate reader, the glucose concentrations in supernatant can be calculated from standard curve.

### Metabolite extraction

For analysis of metabolism, 40 × 10^4^ cells/well SKOV3 cells were seeded onto a six-well plate with basal medium in at least three repeats for 12 h, allowing cells attaching enough. Then steady-state labeling of organic was accomplished by changing the culture medium into glucose-free DMEM medium or glutamine-free RPMI-1640 medium containing appropriate tracer, including 11 mmol/l [U-^13^C_6_] glucose or 2 mmol/l [U-^13^C_5_] glutamine, with or without 0.8 mM TPL for 24 h.

At the end of culture, cells were rinsed twice with normal temperature 0.9% NaCl solution, quenched with 500 μl/well −80 °C methanol. One minute later, 200 μl of ice-cold 5 μg/ml norvaline was added to each well and cells were collected in 1.5 ml Eppendorf tubes by scraping with a pipette. Then 500 μl of −20 °C chloroform was added to each tube followed by vortexing for 15–20 min, and centrifugation at 14,000 × *g* for 10 min at −4 °C. The upper aqueous phase and the lower organic layer were transferred to the fresh tube and exsiccated of airflow, respectively. These dried sample can be stored at −20 °C.

### Metabolite derivation

Derivation should be done within 24 h before detection. Polar metabolites were derivatized to form methoxime–tBDMS derivatives by dissolved upper dried metabolites with 20 μl of 2% (m/v) methoxylamine hydrochloride in pyridine and incubating at 37 °C for 60 min. Samples were then silylated by addition of 100 μl of MTBSTFA with 1% tBDMS and incubated at 45 °C for 30 min. Transferred to glass GC vials for analysis.

### GC–MS analysis

Derivatized metabolites were analyzed using a Thermo GC 1300 connecting to a Thermo MS ISQ. For polar metabolites, the GC oven temperature was held at 100 °C for 2 min, and increased to 255 °C at 3.5 °C/min followed by increasing to 320 °C at 15 °C/min and held for 3 min. Electron impact ionization was operated with the MS scanning over the range 100–650 *m*/*z*.

### Determination of extracelluler acidification and oxygen consumption

Then measurement of extracelluler acidification rate (ECAR) and oxygen consumption rate (OCR) were detected by XF96 Extracellular Flow analyzer (Seahorse Bioscience, Billerica, Ma, USA) in real time. Briefly, 1 × 10^4^ cells/well SKOV3 cells were cultured in custom XF96 microplates. After cell attaching well (~12 h), the media was replaced with 80 μl of RPMI-1640 media containing indicated concentration of TPL for 24 h. Before measurement, non-buffer XF assay medium was preheated to 37 °C, and cells were washed with the assay medium twice. Immersed in 180 μl non-buffered medium and incubated in the 37 °C, and non-CO_2_ incubator for 1 h. All experiments were performed at 37 °C. In order to eliminate the effect of the cell proliferation rate, determined the concentrations of protein in each well after finishing the assay and then normalized either OCR or ECAR by dividing the concentration. Cellular viabilities that were determined after assay were nearly indistinguishable regardless of 24 h TPL exposure or not. For measurement of ECAR, glucose (10 mM), oligomycin (1 μM), and 2-DG (50 mM) were added at a specified point in time. For measurement of OCR, oligomycin (1 μM), FCCP (0.5 μM), and rotenone-antimycin A (0.5 μM) were added at a specified point in time. The bracketed concentrations of drugs are the final concentrations.

### CCK 8 assay

A total of 3000 cells/well SKOV3 cells were cultured on 96-well plate. After cells attached on the wells, the medium was exchanged into glutamine-free RPMI-1640 medium, glutamine-free RPMI-1640 medium containing 0.8 mM TPL, glutamine-free RPMI-1640 medium containing 2 mM α-ketoglutarate, glutamine-free RPMI-1640 medium containing 2 mM α-ketoglutarate, and 0.8 mM TPL to culture for 48 h. Washed cells with glutamine-free RPMI-1640 medium twice, following by adding 100 μl glutamine-free RPMI-1640 medium to each well. Then 10 μl of CCK8 was added to each well and the cells were incubated at 37 °C for 2 h away from light. The absorbance was read at 450 nm using Bio-Tek multi-mode reader.

### Molecular modeling

Autodock vina docking software, offered free by The Scripps Research Institute (http://vina.scripps.edu/), was used to predict the binding mode. The three-dimensional (3D) structure of TPL was obtained from PubChem database (https://pubchemdocs.ncbi.nlm.nih.gov/). The resulting structures were saved in 3D SDF format. The crystallographic structures of isocitrate dehydrogenase 1 (IDH1) (PDB ID: 6ADG) and IDH2 (PDB ID: 5198) were obtained from Protein Data Bank (https://www.rcsb.org/). To prepare the structure for docking, the structure of ligand and proteins were changed into pdbqt format. Autodock vina software was utilized for docking in this study. Optimize the interaction between ligands and receptors via semiflexible docking. In general, the docking parameters remain Vina default but the number of Autodock vina runs was 100 times.

### Western blot analysis

Cell were cultured with the medium containing 0.8 mM TPL or not for 24 h and rinsed quickly with PBS. Then the cells were lysed with RIPA lysis buffer with 1% protein phosphatase inhibitors (Fdbio science, Hangzhou, China) and 1% PMSF (Fdbio science, Hangzhou, China). Protein content was measured using BCA protein kit (Beyotime, Shanghai, China). For measuring the expression level and phosphorylation level of IDH1/IDH2, 10 μg of cell lysates were separated by 10% SDS–PAGE and 10% phosbind SDS–PAGE (Apexbio, Houston, TX, USA) respectively. The phosbind SDS–PAGE was washed gently in protein transfer buffer with 5 mmol/l EDTA for three times and general protein transfer buffer once. Then the samples were transferred to PVDF membrane, and blocked in 5% BSA. PVDF membranes were incubated in appropriate primary antibodies overnight at 4 °C. These antibodies were anti-IDH1 antibody (Abcam, Cambridge, UK, 1:5000 dilution), anti-IDH2 antibody (Abcam, Cambridge, UK, 1:5000 dilution), and anti-GAPDH antibody (Proteintech, Chicago, IL, USA, 1:10,000 dilution). The membranes were washed with TBST and then incubated with HRP-conjugated secondary antibodies (Bioworld, Minneapolis, MN, USA, 1:10000 dilution) for 1 h. The bands were scanned using Bio-Rad molecular imager.

### Enzyme assays

The activities of IDH1 and IDH2 were detected using Isocitrate Dehydrogenase Activity Colorimetric Assay Kit according to the manufacturer’s instructions (BioVision, SanFrancisco, CA, USA). Briefly, recombinant human IDH1 (Abcam, Cambridge, UK) and IDH2 (Abcam, Cambridge, UK) were diluted with IDH assay buffer, and added into 96-well plate. And indicated concentration of TPL were added to these wells. Adjust the final volume to 50 µl with assay buffer. A total of 50 µl of the reaction mix, containing NADP^+^, IDH substrate, and developer, was added to each well. The mix was incubated for 3 min at 37 °C. The OD 450 nm was measured in a Bio-Tek multi-mode reader, and then the absorbance was read at 450 nm every 10 min, for 90 mins. The mean values of background wells without enzyme were subtracted from all readings.

### Animals

Female BALB/c nude (nu/nu) mice (5–8-week old) were obtained from the Animal Experimental Center of Guangdong (China). The mice were house in a climate controlled and control 12-h circadian rhythm-adjusted room, and were allowed access to food and water ad libitum. All animal experiments in this study were approved by the Medical Ethics Committee of Southern Medical University.

### Tumor and mice models

SKOV3 cells were implanted subcutaneously (s.c.) in BALB/c nude (nu/nu) mice at a concentration of 2 × 10^6^ in both flanks. Tumor size was assessed every 2 days by caliper measurements, using the formula width^2^ × length × 0.5. Mice received intraperitoneal (i.p.) drug treatment when tumors reached 50–100 mm^3^. The mice were randomly divided into two groups: CON (saline, *n* = 9) and TPL (1.6 mmol/kg, daily, *n* = 9). All animals were weighed every day.

### Hematoxylin and eosin staining

Mouse kidneys and livers were harvested 14 days after start of therapy, and fixed in 4% paraformaldehyde for 24 h, then gradually dehydrated and embedded in paraffin. The samples were cut into 3-μm sections and stained with hematoxylin and eosin (H&E) for general histological examination.

### Immunohistochemistry

Tumors were harvested 21 days after start of therapy. Tumors were fixed in 4% paraformaldehyde and embedded in paraffin. A total of 3-μm thick sections were stained for Ki-67 (Abcam, Cambridge, UK, 1:300 dilution) followed by HRP-conjugated secondary antibody using diaminobenzidine reagents and then counterstained with hematoxylin. Negative control was performed by omitting primary antibody. Five fields per tumor were randomly selected at a magnification of ×200 and ×400. The percentage positive cells were determined for each field.

### Tunel assay

The apoptosis detection in tumor tissue were performed using TUNEL apoptosis detection kit according to the manufacturer’s instructions (YEASEN, Shanghai, China). A total of 3-μm thick sections were dewaxed with xylene and gradually dehydrated with concentration gradient alcohol. Tissue sections were incubated with 2 mg/ml proteinase K for 20 min at RT. Then each sample was kept in 100 μL 1× equilibration buffer for 30 min. A total of 2 μg/ml DAPI was used to stain samples for 5 min after incubating with 50 μl TdT incubation buffer for 60 min at 37 °C away from light, and examined using a fluorescent microscope.

### Statistical analysis

Results are presented as mean ± standard deviation. For pairwise comparisons, statistical analysis was performed using the Student’s *t*-test, and the statistical analyses were performed using GraphPad Prism statistical software (GraphPad Software, Inc., San Diego, CA, USA). Differences in tumor growth at specific time points were analyzed by one-way ANOVA, and these analyses were performed with the SPSS statistical software (SPSS, Inc. Chicago, IL, USA). *P* value < 0.05 was considered statistically significant. Statistical significance was defined as *P < 0.05; **P < 0.01; ***P < 0.001; no sign, no significant difference.

## Results

### TPL treatment reduces ROS level and inhibits mitochondrial OXPHOS

**Fig. 1:**
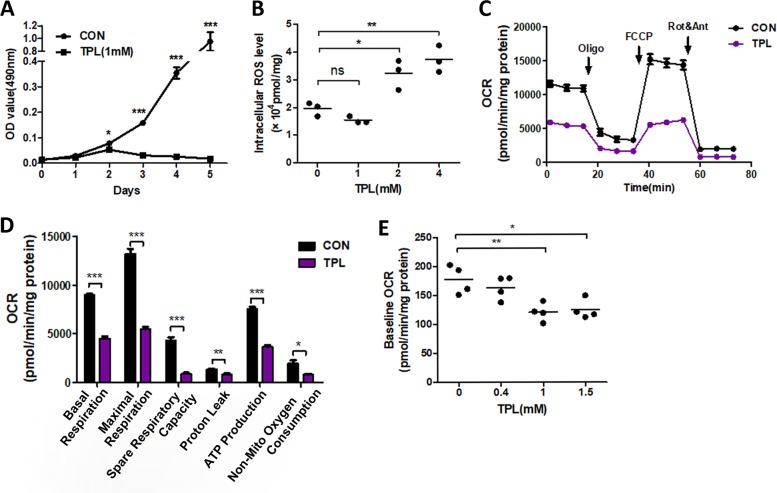
TPL treatment inhibited mitochondrial OXPHOS and reduced ROS level. **a** MTT assay of SKOV3 cells treated with 1 mM TPL and control. **b** Effect of TPL in different concentrations on intracellular ROS production from drug treatment for 24 h by DCFH-DA-dependent measurements using flow cytometry analysis. **c**, **d** SKOV3 cell were treated with control (black) or 0.8 mM TPL (purple) for indicted time before measurement of OCR detected by Seahorse XFe96 Extracellular Flow analyzer in real time. Arrow is the time of inhibitors adding sequential. Oligomycin (1 μM), FCCP (0.5 μM), and rotenone-antimycin A (0.5 μM). The bracketed concentrations of drugs are the final concentrations. Histogram of OCR rate was shown **d**. **e** The baseline OCR of SKOV3 cells after treating with indicated concentration of TPL for 12 h. For all experiments, values are mean ± SD carried out in triplicate. *P < 0.05; **P < 0.01; ***P < 0.001; no sign means no significant difference; Student’s *t*-test.

We detected the cell proliferation after the treatment of TPL and we observed that 1 mM TPL treatment for 48 h significantly reduced cell proliferation (Fig. 1a), while cellular morphology hasn’t observable change (data not show). ROS is critical for the effectiveness of chemotherapeutic drugs. We analyzed cellular ROS by using emission fluorescence intensity in cells incubated in the presence of DCFH-DA and found that cellular ROS level was increased in a concentration-dependent matter when the concentration of TPL was >2 mM (Fig. 1b), while the ROS level was not increased by lower concentration TPL treatment (1 mM), suggesting that the effect of TPL on ROS production in cancer cells depended on the concentration of TPL (Table 1).

**Table 1:** Docking results of TPL on both IDH1 and IDH2.

**Protein**	**PDB ID**	**Tempol banding** energy(kcal/mol)
IDH1	6ADG	−8.9
IDH2	5198	−12.7

Mitochondrial is a major source of ROS, and TPL was reported to reduce the cellular OXPHOS in zebrafish^29^. To determine the effect of TPL on OXPHOS in cancer cells, we used extracellular flux analyzer Seahorse XF96e to measure the OCR. After the 24-h treatment of TPL, basal mitochondrial OCR was significantly reduced in SKOV3 (Fig. 1c, d). After treating with oligomycin and protonophoric uncoupler FCCP, both maximal and reserve mitochondrial capacities were significantly reduced in the presence of TPL. Besides, the proton leak and the non-mito oxygen consumption (using the electron transport inhibitor rotenone) were reduced by TPL treatment. Next, we evaluated the baseline of OCR in cells treated with indicated concentration of TPL and showed that the baseline of OCR was significantly reduced (Fig. 1e), in line with the above result. These results indicated that TPL treatment inhibited OXPHOS of SKOV3 cells.

**Fig. 2:**
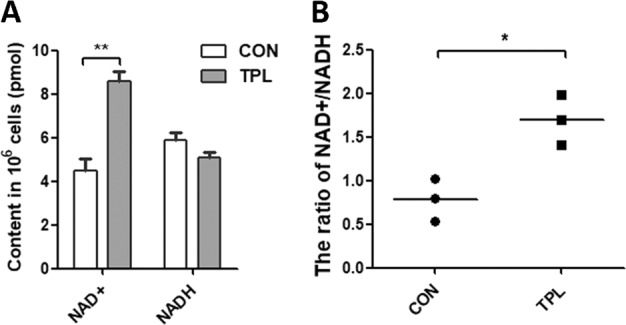
TPL treatment increased the ratio of NAD^+^/NADH in cells. **a** The content of NAD^+^ and NADH in cells after treating with 0.8 mM TPL or not. **b** The ratio of NAD^+^/NADH in cells after treating with 0.8 mM TPL or not (*n* = 3). For all experiments, values are mean ± SD carried out in triplicate. *P < 0.05; **P < 0.01; ***P < 0.001; no sign means no significant difference; Student’s *t*-test.

We then detected the content of NADH that is generated mainly from the TCA cycle and donates electrons for complex I as part of OXPHOS in cells. The data showed that TPL treatment significantly increased the content of NAD^+^ and elevated the ratio of NAD^+^/NADH (Fig. 2a, b), suggesting that TPL treatment inhibited the production of NADH from the TCA cycle.

### The effect of TPL on glycolysis in cancer cells

**Fig. 3:**
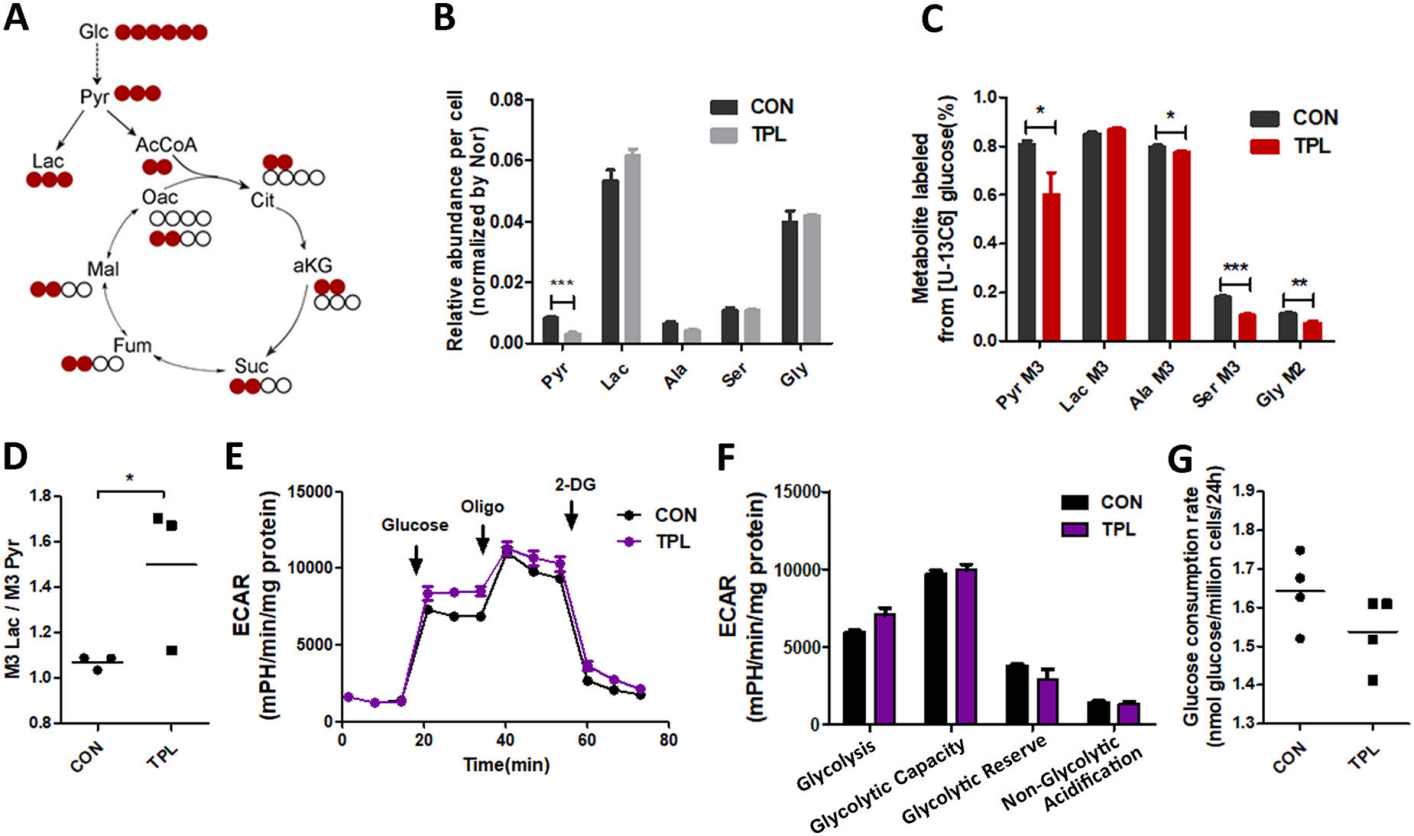
TPL reduced the production of pyruvate and inhibited the serine pathway. **a** Schematic map of carbon atom (represented by circles) transitions and tracer used to detect glucose metabolism, [U-^13^C_6_] glucose (red). **b** Relative abundance of pyruvate, lactate, and three amino acids (alanine, glycine, and serine) per cell after maintaining in [U-^13^C_6_] glucose medium that contained 0.8 mM TPL (gray) or not (black) for 24 h (*n* = 3). **c** Relative level of glycolysis and serine pathway, determined by M3 or M2 labeling of metabolites from [U-^13^C_6_] glucose in control (black) and TPL group (red; *n* = 3). **d** The ratios of M3 lactate/M3 pyruvate. **e**, **f** SKOV3 cell were treated with control (black) or 0.8 mM TPL (purple) for indicted time before measurement of ECAR were detected by Seahorse XFe Extracellular Flow analyzer in real time (*n* = 3). Arrow indicate the time of inhibitors adding sequential. Glucose (10 mM), oligomycin (1 μM), and 2-DG (50 mM); the bracketed concentrations of drugs are the final concentrations. Histogram of ECAR rate was shown **f**. **g** Glucose consumption rate in SKOV3 cells after treated with control (white) or TPL (black) for 24 h (*n* = 4). For all experiments, values are mean ± SD carried out in triplicate. *P < 0.05; **P < 0.01; ***P < 0.001; no sign means no significant difference; Student’s *t*-test.

The disturbance of the TCA cycle is an important cause of mitochondrial respiration inhibition. Glycolysis is critical for generating pyruvate to fuel TCA. To determine whether TPL influences glycolysis of cancer cells, we did GC–MS-based^13^C metabolic flux analysis to analyze metabolic fluxes. Mass isotopomer distributions represent the relative abundance of ion fragments with a different number of ^13^C (ref. ^30^). Mass isotopomers M0, M1, M2, etc. refers to the ion fragments with zero, one, or two ^13^C, respectively. The steady levels of metabolite and the metabolic pathways by testing the ^13^C-labeled metabolites enable us to analyze metabolic flux distribution (Fig. 3a). SKOV3 cells maintained in media with [U-^13^C_6_] glucose was treated or not with 0.8 mM TPL for 24 h and then were analyzed with GC–MS. The TPL-treated cells exhibited a threefold decrease of total pyruvate production, but no change of total lactate and three amino acids (alanine, glycine, and serine) amount (Fig. 3b). We found that glucose-derived M3 pyruvate, M3 alanine, M3 serine, and M2 glycine but not M3 lactate were significantly reduced in TPL-treated cells when we measured the metabolites labeled with ^13^C (Fig. 3c). The result indicated that TPL inhibited pyruvate production and serine pathway.

We further evaluated the ECAR and showed that TPL treatment had no effect on extracellular lactate derived from glycolysis and from non-glycolysis (Fig. 3e, f). Our data shows the conversion from pyruvate to lactate increased in TPL-treated cells (Fig. 3d). We also measured glucose consumption of SKOV3 cells treated with TPL by using Glucose Colorimetric Assay. The result showed that TPL treatment didn’t change glucose consumption (Fig. 3g). Taken all together, the data suggested that TPL treatment had less effect on glycolysis but reduced the production of pyruvate and inhibited the serine pathway.

### TPL interferes with glutamine metabolism by inhibiting conversion between isocitrate and α-ketoglutarate

**Fig. 4:**
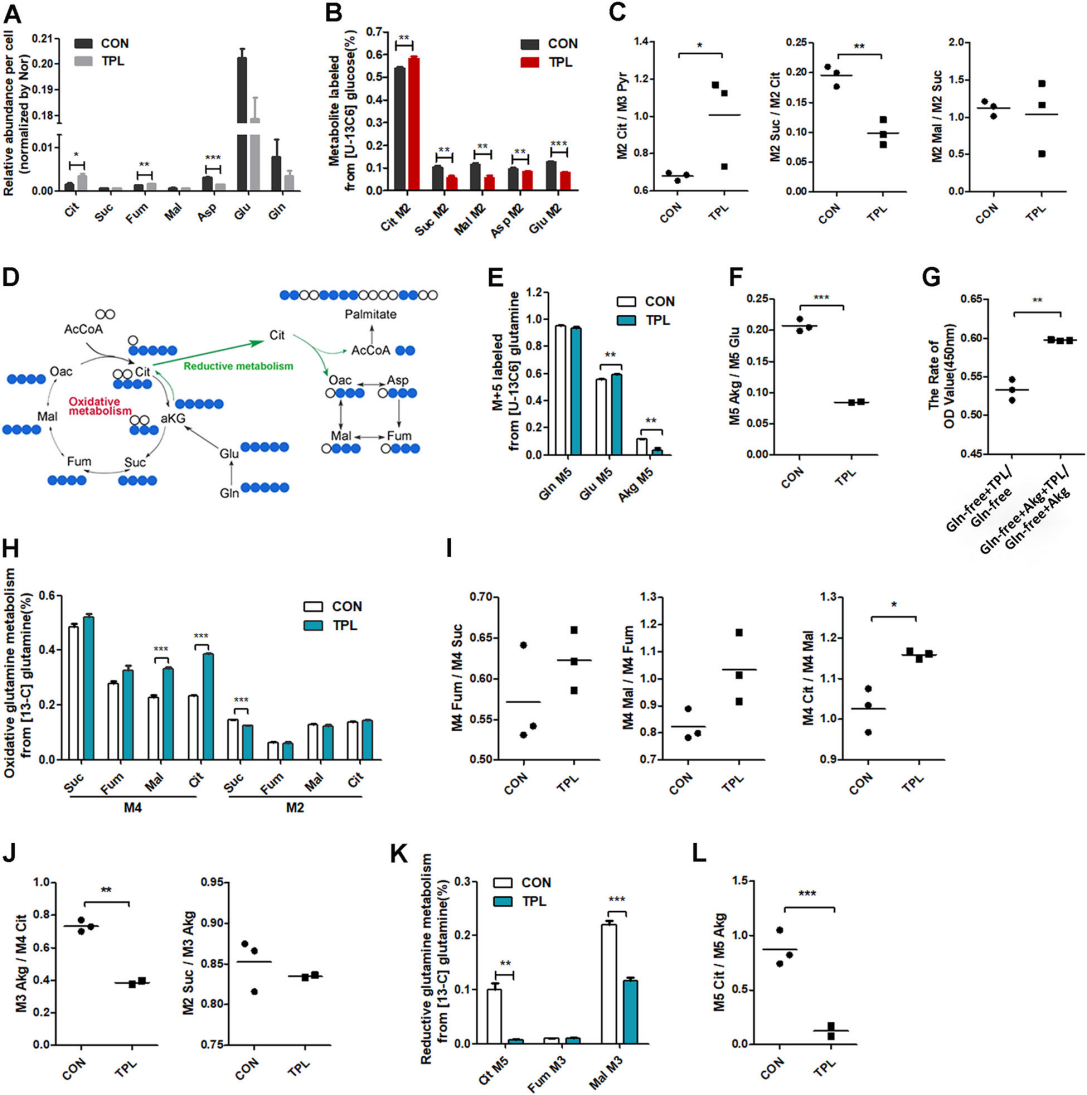
TPL blocked conversion between α-ketoglutarate and citrate. **a** Relative abundance of TCA cycle intermediates and three TCA-related amino acids per cell after the cell treated as in Fig. 3b (*n* = 3). **b** Relative level of TCA cycle and amino acid, determined by M2 labeling of metabolites from cells cultured as in Fig. 3b (*n* = 3). **c** The ratios of M2 citrate/M3 pyruvate, M2 succinate/M2 citrate, and M2 malate/M2 succinate from cells cultured as in Fig. 3b. **d** Carbon atom (represented by circles) transitions and tracer used to detect changes in flux, [U-13C5] glutamine (blue). **e** Relative level of M5 glutamine, glutamate, and α-ketoglutarate from [U-13C5] glutamine after 0.8 mM TPL treatment or not for 24 h. TPL (blue), control (white; *n* = 3). **f** The ratios of M5 α-ketoglutarate/M5 glutamate from the cells cultured as in **e**. **g** The rate of OD value (450 nm) of Gln-free + TPL/Gln-free and Gln-free + Akg + TPL/Gln-free + Akg. Gln-free represents RPMI-1640 medium without glutamine (*n* = 3, Akg represents α-ketoglutarate). **h** Relative level of oxidative glutamine metabolism, determined by M4 and M2 labeling TCA cycle metabolites from [U-13C5] glutamine and the cell cultured as in **e** (*n* = 3). **i**, **j** The ratios of M4 fumarate/M4 succinate, M4 malate/M4 fumarate, M4 citrate/M4 malate, M3 α-ketoglutarate/M4 citrate, and M2 succinate/M3 α-ketoglutarate. **k** Relative level of reductive glutamine metabolism, determined by M5 citrate, M3 fumarate, and M3 malate from the cells cultured as in **e** (*n* = 3). **l** The ratios of M5 citrate/M5 α-ketoglutarate. For all experiments, values are mean ± SD carried out in triplicate. *P < 0.05; **P < 0.01; ***P < 0.001; no sign means no significant difference; Student’s *t*-test.

We further explored the effect of TPL on cellular metabolism, we analyzed the relative abundance of TCA cycle metabolites derived from glucose. Some TCA cycle intermediates in TPL-treated cells increased significantly and three TCA-related amino acids (aspartate, glutamine, and glutamate) reduced (Fig. 4a). TCA cycle intermediates labeled with M2 isotopomer metabolites (^13^C_2_ citrate, ^13^C_2_ α-ketoglutarate, ^13^C_2_ malate, and ^13^C_2_ glutamate) were derived from U-^13^C_6_ glucose. The percentage of M2 citrate was significantly increased, but the M2 glutamate (derived from M2 α-ketoglutarate), M2 malate, and M2 aspartate (derived from M2 oxaloacetate) were all significantly reduced in TPL-treated SKOV3 cells (Fig. 4b). The ratio of M2 citrate to M3 pyruvate increased significantly, but M2 succinate/M2 citate decreased and not in the M2 malate/M2 succinate ratio after TPL treatment (Fig. 4c), suggesting reducing the effect of TPL on the oxidation procession from citrate to succinate. The above results indicated that TPL inhibited TCA in the glucose metabolism of cancer cells.

We further investigated the effect of TPL on the TCA cycle by GC–MS metabolic flux analysis with [U-^13^C_5_] glutamine (Fig. 4d). In glutaminolysis, glutamine is converted into glutamate and then oxidized into α-KG for TCA metabolism. The abundance of M5 glutamine didn’t change in TPL-treated cells (Fig. 4e), indicating TPL didn’t increase the uptake of glutamine. The M5 glutamate abundance increased while the M5 α-ketoglutarate reduced significantly, leading to a significant reduction for the ratio of M5 glutamate/M5 α-ketoglutarate (Fig. 4f). Furthermore, we examined the effect of TPL on cell viability when SKOV3 cells were cultured with glutamine-free medium supplementation with α-ketoglutarate. The rate of Gln-free + Akg + TPL/Gln-free + Akg had an increase compared with the rate in the medium without α-ketoglutarate (*P* < 0.05, Fig. 4g), indicating that the inhibitory effect of TPL on cell viability was partially rescued after α-ketoglutarate supplementation. This result suggests that inhibition of either transaminases or GDH and conversion of glutamate to α-ketoglutarate was impeded after TPL treatment.

After entering to TCA cycle, M5 α-ketoglutarate converted into succinate, fumarate, malate, and citrate labeled with M4 isotopomer metabolites (^13^C_4_ succinate, ^13^C_4_ fumarate, ^13^C_4_ malate, and ^13^C_4_ citrate). As showed in Fig. 4h, TPL induced an increase of M5 glutamine-derived oxidative intermediates M4 malate, and M4 citrate, but the reduction of M2 succinate from M4 citrate (Fig. 4h). The ratio of M3 α-ketoglutarate/M4 citrate was reduced but the ratio of M4 fumarate/M4 succinate, M4 malate/M4 fumarate, M4 citrate/M4 malate, and M2 succinate/M3 α-ketoglutarate didn’t change or even slightly increased (Fig. 4i, j). Accumulation of M4 intermediates may explain the impeding procession of citrate to α-ketoglutarate.

Glutamine can also generate acetyl-CoA via reductive carboxylation, wherein glutamine-derived α-ketoglutarate is carboxylated to produce isocitrate/citrate, which is then cleaved to produce oxaloacetate and acetyl-CoA. In the GC–MS FMA assay, [U-^13^C_5_]glutamine-derived M5 α-ketoglutarate is carboxylated to produce to M5 citrate and further decarboxylated to other 4-carbon intermediates (M3 malate, M3 fumarate, M3 oxaloacetate, and M3 aspartate). The abundance of the M5 citrate was dramatically reduced in TPL-treated SKOV3 cells and M3 malate also reduced onefold (Fig. 4k). To eliminate the effect of the decrease abundance of α-ketoglutarate, we evaluated the amount of M5 citrate by normalizing the amount of M5 α-ketoglutarate in both groups. The ratio of M5 citrate/M5 α-ketoglutarate was significantly decreased after TPL treatment (Fig. 4l), suggesting that TPL blocks the conversion of α-ketoglutarate to isocitrate. Taken it together, the results indicated that TPL interferes with glutamine metabolism by blocking conversion between isocitrate and α-ketoglutarate.

### TPL inhibited the activity of IDH1 and IDH2 directly in SKOV3 cells

**Fig. 5:**
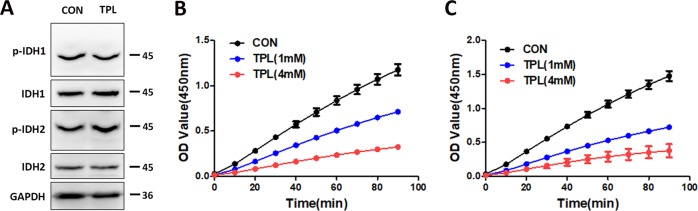
TPL inhibited the IDH1 and IDH2 enzyme activity. **a** The effects of TPL on the phosphorylation of IDH1 and IDH2 in SKOV3 cells were analyzed by a Phos-tag assay. Immunoblots for total IDH1 and IDH2 were also performed on normal SDS–PAGE gels. **b**, **c** Enzymatic activity of both IHD1 and IDH2 treated with control and TPL (*n* = 3). For all experiments, values are mean ± SD carried out in triplicate. *P < 0.05; **P < 0.01; ***P < 0.001; no sign means no significant difference; Student’s *t*-test.

Conversion between citrate and α-ketoglutarate is a two-step reaction, including the conversion of citrate-to-isocitrate and isocitrate-to-α-ketoglutarate. The conversion of isocitrate-to-α-ketoglutarate is a key step that is catalyzed by IDHs, most frequently mutated proteins in cancer^31,32^. There are three IDH isoforms, of which IDH1 and IDH2 catalyze either oxidative decarboxylation or reduction carboxylation^33,34^. To investigate whether TPL inhibited conversion between α-ketoglutarate and citrate by inhibiting IDH1/2 expression and phosphorylation, we performed western blotting. The result showed that the TPL treatment didn’t change IDH1/2 expression and phosphorylation (Fig. 5a).

To clarify the ability of TPL binding to the target protein and identify the binding mode, we conducted docking simulations for TPL to IDH1/2 by applied Autodock vina. The result shows the binding energies of TPL to IDH1 and IDH2 are −8.9 and −12.7 kcal/mol, respectively (Table 1). We then determined the activities of recombinant IDH1/2 by detection of the increase in NADPH concentration. The results showed that TPL treatment directly inhibit the activity of purified recombinant IDH1/2 protein. These results indicated that TPL inhibited IDH1/2 activity directly but not inhibiting IDH1/2 expression and phosphorylation (Fig. 5b, c).

### TPL inhibited the growth of SKOV3 cells in xenograft animal model

**Fig. 6:**
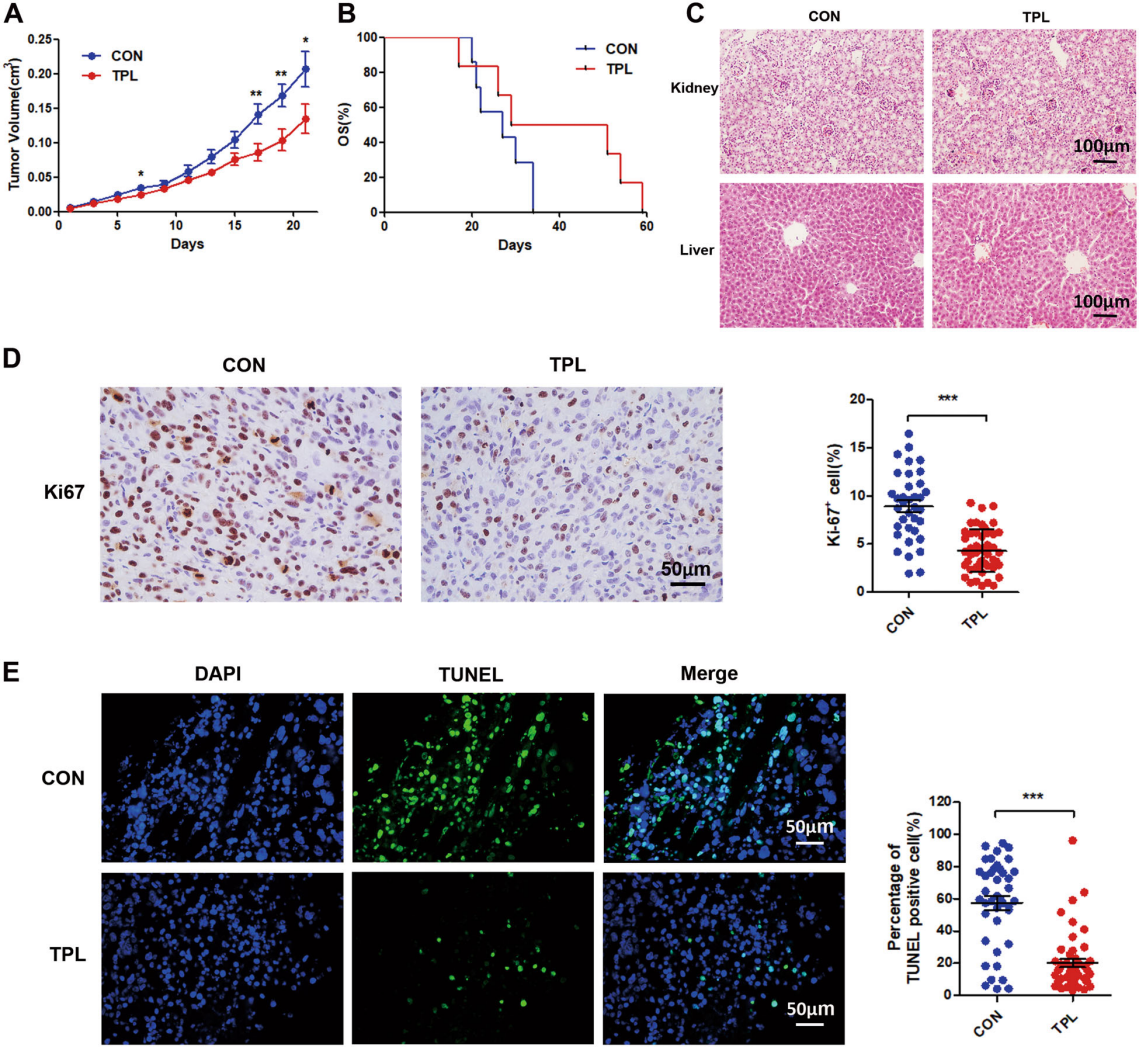
TPL inhibited tumor growth and proliferation of tumor cell in xenograft animal model. **a**, **b** Time course and survival curves of SKOV3 tumor growth in control (blue, 0.9% NaCl 0.4 ml/25 mg, i.p., daily) and TPL-treated (red, TPL 1.6 mmol/kg, i.p, once a day,7 days/week) mice (*n* = 6–9 per group). **c** The H&E staining of kidneys and liver from mouse in both treatments (scale bars, 100 μm). **d** Representative images of Ki-67 staining of tumor tissues (scale bars, 50 μm) from mouse in both treatments, and association analyses of the number of Ki-67 + cells in tumor tissues. **e** The representative image of fluorescent DAPI and TUNEL staining of xenograft tumor sections, and association analysis of number TUNEL positive cells (scale bars, 50 μm). Data are the mean ± SD for experiments. *P < 0.05; **P < 0.01; ***P < 0.001; no sign means no significant difference; Student’s *t*-test.

To examine whether TPL has antitumor activity in a mouse model, we established a xenograft mouse model by s.c. injection of SKOV3 cells to BALB/c nude mice. We applied a dose of TPL to 1.6 mmol/kg, a significant inhibition of tumor growth was observed and the lifespan was prolonged although it’s not significant in statistics (Fig. 6a, b), indicating that TPL treatment inhibiting tumor growth in xenografted mice. The TPL-treated group also displayed no effect on body weight and histo-morphology of kidney and liver analyzed by HE staining (Fig. 6c). The cell proliferation was detected by the expression of Ki-67 in tumor tissues. The percentages of the Ki-67-positive cell in tumor tissues decreased in TPL-treated mice (Fig. 6d).

The elevated oxidative stress is an important inducer of apoptosis. We then detected whether TPL had an inhibitory effect on tumor cell apoptosis in the mouse model. TUNEL assay showed that TPL markedly reduced the number of apoptotic cells compared with control (Fig. 6e). These results demonstrate that the inhibitory effect of TPL on tumor growth was not by inducing cell apoptosis, but by inhibiting cell proliferation in vivo in the xenograft tumor model.

## Discussion

TPL has been reported to induce cellular apoptosis of many types of cancer cells and inhibit tumor growth in immune-defective mice^35,36^. In this study, we confirmed that the cytotoxicity of TPL was dependent on high concentration and induced cellular apoptosis that associated to increase in cellular ROS. However, the dosage of TPL used in vivo inhibited cellular proliferation but not increase the events of cellular apoptosis. The concentration of TPL in the tumors of xenograft mice cannot reach the concentration of inducing ROS, so it may work as an antioxidant in vivo. Here, we proposed a new mechanism of TPL inhibiting cancer cell proliferation by interfering with glutamine metabolism.

Cellular proliferation is closely associated with metabolism. The rapid proliferation of cancer cells requires a large number of macromolecular precursors and NADPH produced from glycolysis and TCA cycle^33,37,38^. It is well known that, in addition to glucose, glutamine is also an important nutrient for tumor cell growth and viability^23^. Glutamine is a conditionally essential amino acid, and can provide nitrogen and carbon for the TCA cycle, fatty acid synthesis and nucleotide synthesis^34,39,40^. Glutamine metabolism includes the catabolic process (glutaminolysis) and anabolic process (reductive carboxylation), which are vital for many cancer cells to maintain survival. Glutaminolysis can fuel the oxidative TCA cycle flux, which is important for sustaining ATP production and redox homeostasis in cells, especially when glucose preferentially enters the process of aerobic glycolysis in cancer cells^41–43^. Glutamine also provides the major source of acetyl-CoA for lipid synthesis via reductive carboxylation, which significantly reduces the need for acetyl-CoA derived from glucose. Furthermore, the citrate produced by the reductive carboxylation is cleaved to oxaloacetate, which can be converted to other 4-carbon intermediates to complement the TCA cycle^22,44^. Besides, it is reported that blocking glutamine can relieve the immunosuppressive effect of the tumor microenvironment, and at the same time can enhance the activity of T cells^45^. Hence, disturbing glutamine metabolism can be a strategy for cancer treatment.

Here, we found that TPL had no significant effect on glycolysis although it lowered the production of pyruvate and intermediates of the serine pathway. However, TPL not only blocked the generation of α-ketoglutarate from glutamate but also significantly interfered with both glutaminolysis and reductive carboxylation processes via the inhibition of conversion between citrate/isocitrate and α-ketoglutarate, which are catalyzed by IDHs.

There are three isoforms of IDH in a human cell: IDH1, IDH2, and IDH3. IDH3 uses NAD^+^ as cofactor and functions exclusively as oxidative decarboxylase. IDH1 and IDH2 catalyze either oxidative decarboxylation or reduction carboxylation using NADP^+^/NADPH as cofactors^34,43^. Recent studies suggest that IDH1 and IDH2 mutations exist in a variety of tumor types, such as glioma, chondrosarcoma, and acute myeloid leukemia^46^. Besides, the aberrant expression of wild-type IDHs is also found in some tumors. Overexpression of IDHs is associated with the poor overall survival of cancer patients^47–49^. Our results suggested that the inhibition of TPL on activities of IDH1/IDH2 to interfere with the interconversion between citrate and α-ketoglutarate in TCA cycle is one important mechanism of TPL disturbing cellular metabolism. Blocking citrate metabolism by TPL led to the citrate accumulation and downstream metabolites reduction. First, citrate is an allosteric inhibitor of phosphofructokinase (PFK), a key enzyme regulating glycolysis flow in tumor cells^50^. Accumulation of citrate inhibits the activity of PFK, which may explain reduced glucose-derived pyruvate production and inhibited serine pathway in TPL-treated cells. Second, the TCA cycle generates a large number of NADH/FADH_2_, which provides electrons for OXPHOS (ref. ^51^). TPL inhibited the conversion of citrate to α-ketoglutarate and disturbed the OXPHOS at complex I by inhibiting NADH production in cells. These results were consistent with the previous reports, in which TPL impaired complex I in oxidation respiratory chain. Third, TPL perturbing glutamine metabolism could further inhibit the TCA cycle and lipid synthesis, which may explain the reduced cellular proliferation and slower growth of tumors after TPL treatment.

Collectively, TPL exerts multiple mechanisms to influence cell viability. Here, we provided a mechanism of TPL inhibiting cancer cell proliferation by interfering with glutamine utilization, which is important for survival and proliferation of cancer cells. The study may help the development of a therapeutic strategy of TPL combined with other anticancer medicines.
